# A Structured Approach to Teaching Volumetric Fetal Echocardiography Using Fetal Intelligent Navigation Echocardiography (FINE)

**DOI:** 10.1111/echo.70659

**Published:** 2026-10-31

**Authors:** Adriana Ferri Giubilato, Nathalie Jeanne Bravo‐Valenzuela, Maria Elisa Martini Albrecht, André Souza Malho, Edward Araujo Júnior

**Affiliations:** ^1^ Department of Obstetrics, Paulista School of Medicine Federal University of São Paulo (EPM‐UNIFESP) São Paulo SP Brazil; ^2^ Department of Pediatrics, Pediatric Cardiology, School of Medicine Federal University of Rio de Janeiro (UFRJ) Rio de Janeiro RJ Brazil

**Keywords:** fetal intelligent navigation echocardiography, fetal echocardiography, intelligent navigation, spatio‐temporal image correlation, ultrasound education, volumetric ultrasound

## Abstract

Fetal volumetric echocardiography using Fetal Intelligent Navigation Echocardiography (FINE) enables automated reconstruction of standardized echocardiographic views from a cardiac volume dataset. However, automation of navigation does not eliminate dependence on technically adequate acquisition or the need for operator quality control. We describe the PROBE Educational Workflow, a structured educational approach comprising five sequential steps—Position, Refine, Obtain, Build and Evaluate—designed to organize the teaching of the process from preparation of the two‐dimensional image and acquisition of the Spatio‐Temporal Image Correlation (STIC) volume to construction of the navigation and systematic assessment of the reconstructed views. PROBE was conceived as an iterative workflow: when the quality of a step is considered insufficient, the operator should identify the probable origin of the problem and return to the corresponding step before proceeding. The workflow does not modify the FINE algorithm and does not constitute a diagnostic protocol; its purpose is to provide a simple and reproducible cognitive framework for technical training in volumetric fetal echocardiography. Future studies should assess its impact on competency acquisition, volume quality, reproducibility and training efficiency.

## Graphical abstract

PROBE Educational Workflow is a structured educational approach comprising five sequential steps—Position, Refine, Obtain, Build and Evaluate—designed to organize the teaching of the process from preparation of the two‐dimensional image and acquisition of the Spatio‐Temporal Image Correlation (STIC) volume to construction of the navigation and systematic assessment of the reconstructed views.

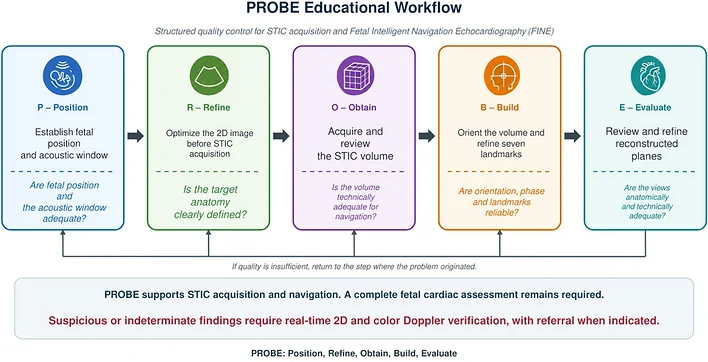

## 1 Introduction

Ultrasonographic assessment of the fetal heart is a core component of prenatal screening. International guidance emphasizes systematic acquisition of recommended cardiac views, image quality and examiner training [1, 2]. Despite technological advances, fetal cardiac assessment remains technically demanding because it involves small, moving structures, complex spatial relationships and substantial dependence on operator skill.

Spatio‐Temporal Image Correlation (STIC) expanded volumetric assessment of the fetal heart by enabling acquisition of datasets containing synchronized spatial and temporal information. These volumes can be reviewed retrospectively and interrogated in multiple planes, providing a basis for volumetric navigation and standardized reconstruction [3, 4].

Fetal Intelligent Navigation Echocardiography (FINE) was subsequently developed to apply intelligent navigation to STIC volume datasets. Following identification of seven anatomical landmarks, the algorithm automatically generates nine standardized echocardiographic views and permits additional exploration using Virtual Intelligent Sonographer Assistance (VIS‐Assistance) [5]. Advanced features of FINE/5D Heart further support automated alignment, cardiac‐phase recognition and targeted refinement of reconstructed planes [6].

Automation simplifies post‐acquisition navigation but does not make the quality of the input volume irrelevant. Prospective studies of FINE have applied adequacy criteria before processing and have emphasized appropriate anatomical inclusion, minimization of relevant acoustic shadowing and motion artifact, and review of the acquired dataset before intelligent navigation [7, 8]. Thus, automation should not be interpreted as the ability to correct a technically inadequate volume.

The educational potential of FINE is also relevant. In non‐expert sonologists, FINE has been associated with higher success in obtaining cardiac views and shorter navigation time than manual navigation [9]. Other studies address STIC acquisition time in FINE Static Mode [10] and compare the quality of standard fetal cardiac views across FAST, STAR and FINE [11]. Recent articles have similarly demonstrated the value of simple, memorable structures for organizing complex fetal cardiac imaging tasks [12, 13].

Against this background, we developed the PROBE Educational Workflow—Position, Refine, Obtain, Build and Evaluate—to organize the teaching of volumetric fetal cardiac acquisition and navigation using FINE. The objective is to make explicit the technical decisions that experienced operators often perform intuitively, while keeping technology quality control distinct from clinical interpretation.

## 2 Description of the Technique: Probe Educational Workflow

PROBE follows the actual chain of image formation and navigation: favorable acquisition conditions are established; the two‐dimensional information is refined; the volume is obtained and reviewed; intelligent navigation is built; and the reconstructed views are evaluated. The five steps and their quality decisions are summarized in Table 1. The workflow is deliberately iterative: when quality is insufficient, the operator should identify where the problem probably originated and either refine the result locally or return to the corresponding previous step. Supplementary Form S1 provides an observer‐rated assessment with explicit performance anchors for the five steps, a separate source‐volume classification and a mandatory safety decision.

**TABLE 1:** Summary of the PROBE educational workflow.

Step	Function	Educational question	Quality decision
P—Position	Establish a favorable fetal position and initial plane	Are fetal position and the acoustic window adequate to begin?	Reposition/adjust the approach before acquisition if necessary
R—Refine	Optimize the two‐dimensional image	Is the anatomical information sufficiently well defined?	Refine image controls before STIC
O—Obtain	Acquire and review the STIC volume	Is the volume technically adequate for navigation?	Proceed to Build when adequate; document and refine a usable but suboptimal volume; return to Position/Refine and reacquire when inadequate or uncertain (Figure 2)
B—Build	Orient the volume and identify or refine the seven anatomical landmarks	Are volume orientation, cardiac phase and all landmarks anatomically reliable?	Confirm or refine the landmarks; return to Obtain when the source volume is inadequate
E—Evaluate	Review and refine the reconstructed diagnostic planes	Are the reconstructed views anatomically consistent and technically adequate?	Accept or update technically adequate planes; return to the source step when quality is insufficient. Persistent suspicion or uncertainty about artifact requires real‐time 2D and color Doppler verification, with referral when indicated (Figure 4).

Abbreviations: STIC, spatio‐temporal image correlation.

For this educational description, the proposed training window is 19–30 weeks' gestation, reflecting the population studied prospectively with FINE [7]. This range has not been validated for PROBE and does not replace the recommended 18–22‐week window for routine fetal cardiac screening [1].

### 2.1 P—Position

Objective: Establish favorable fetal position and an initial four‐chamber view that provide appropriate conditions for volumetric acquisition.

Position occurs before STIC acquisition. The priority is to identify the best available acoustic window and avoid accepting an unfavorable condition merely to advance through the workflow. Published FINE acquisition criteria emphasize sufficient image quality, appropriate anatomical inclusion and minimal interference from relevant acoustic shadowing and motion [7, 8].

The fetal heart should be adequately centered. Maternal habitus, particularly increased abdominal wall thickness, may reduce image quality, while a spine‐anterior fetal position may produce bony shadowing over cardiac structures [1, 7]. These limitations should be recognized before acquisition. When necessary, small changes in transducer position or waiting for a change in fetal position may be more effective than attempting to compensate later for an inadequate dataset.

As a practical pre‐acquisition maneuver within PROBE, the acoustic window can be checked beyond the four‐chamber view by briefly sweeping cranially toward the outflow tracts while maintaining the same general acoustic approach. The goal is to determine whether rib shadowing or another limitation appears over the great vessels before returning to the four‐chamber view for acquisition. Video S1 demonstrates this Position‐to‐Refine sequence.

*Key message*: The quality of FINE begins before STIC acquisition.

### 2.2 R—Refine

Objective: Optimize the two‐dimensional image before volume acquisition.

The information contained in the volume depends on the quality of the two‐dimensional image used as its starting point. The objective is not to prescribe fixed parameter values, but to teach the operator to recognize how image controls affect spatial resolution, temporal resolution, contrast and penetration.

The appropriate fetal cardiac preset should be selected. Depth and magnification should prioritize the cardiac region of interest; focus should be positioned appropriately; gain and dynamic range should preserve anatomical interfaces; and frequency should be adjusted when necessary to balance resolution and penetration. Image‐processing features should improve definition without masking relevant interfaces, while sufficient temporal resolution is maintained.

Frequent technical problems include excessive depth, inappropriate gain, focus outside the cardiac region, unnecessary reduction in temporal resolution and a heart that remains unnecessarily small on the display. The educational principle is straightforward: volumetric acquisition should not begin while the two‐dimensional image can still be technically improved. Video S1 and Figure 1 demonstrate the combined Position and Refine steps and the transition to volume acquisition.

**FIGURE 1:**
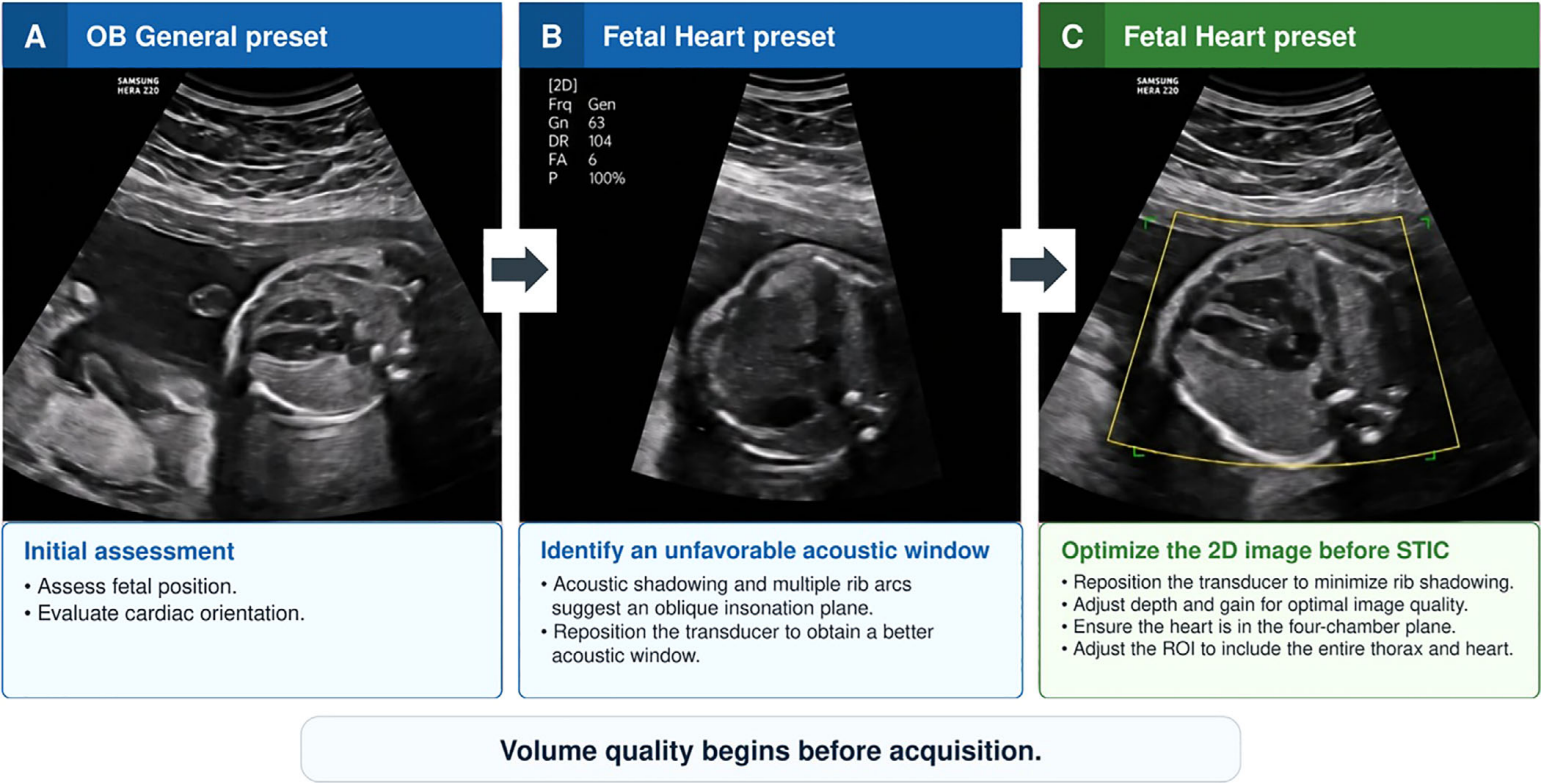
Position and Refine before STIC acquisition. (A) Initial assessment of fetal position and cardiac orientation using the OB General preset. (B) After switching to the Fetal Heart preset, acoustic shadowing and multiple unequal rib arcs may suggest an unfavorable or oblique insonation plane. (C) After transducer repositioning and 2D optimization, the heart is centered in the four‐chamber view and the ROI encompasses the fetal thorax and heart, ready for STIC acquisition. The rib‐arc appearance is a practical teaching cue, not an independently validated diagnostic or adequacy criterion. ROI, region of interest; STIC, spatiotemporal image correlation.

*Key message*: Image optimization cannot be replaced by post‐processing.

### 2.3 O—Obtain

Objective: Acquire a technically adequate STIC volume and verify its quality before applying FINE.

After Position and Refine, volumetric acquisition is activated. The acquisition angle and sweep time should be adapted to gestational age, the anatomical region to be included, fetal position and fetal motion rather than treated as fixed PROBE settings. The transducer should remain stable during the sweep, and maternal or fetal movement that could impair spatial coherence should be minimized. Brief maternal breath suspension may be useful when appropriate.

Acquisition does not end when the sweep is completed. The dataset should be reviewed in STICLOOP before FINE processing. Published criteria include appropriate anatomical inclusion, minimal relevant acoustic shadowing and absence or minimal presence of motion artifact [7, 8]. The review should also look for discontinuity between adjacent planes, loss of alignment and other evidence of spatial distortion.

In the workflow demonstrated in Video S2 and Figure 2, automatic STICLOOP playback is paused and the dataset is manually scrolled through the acquired planes to assess spatial coherence. As a practical teaching cue, the rib arcs can be followed across adjacent planes: continuous and consistent alignment supports coherence, whereas abrupt loss of continuity or the appearance of multiple unequal rib arcs should prompt closer reassessment for obliquity, misalignment or motion‐related distortion. This cue is an educational aid and has not been independently validated as an adequacy criterion.

**FIGURE 2:**
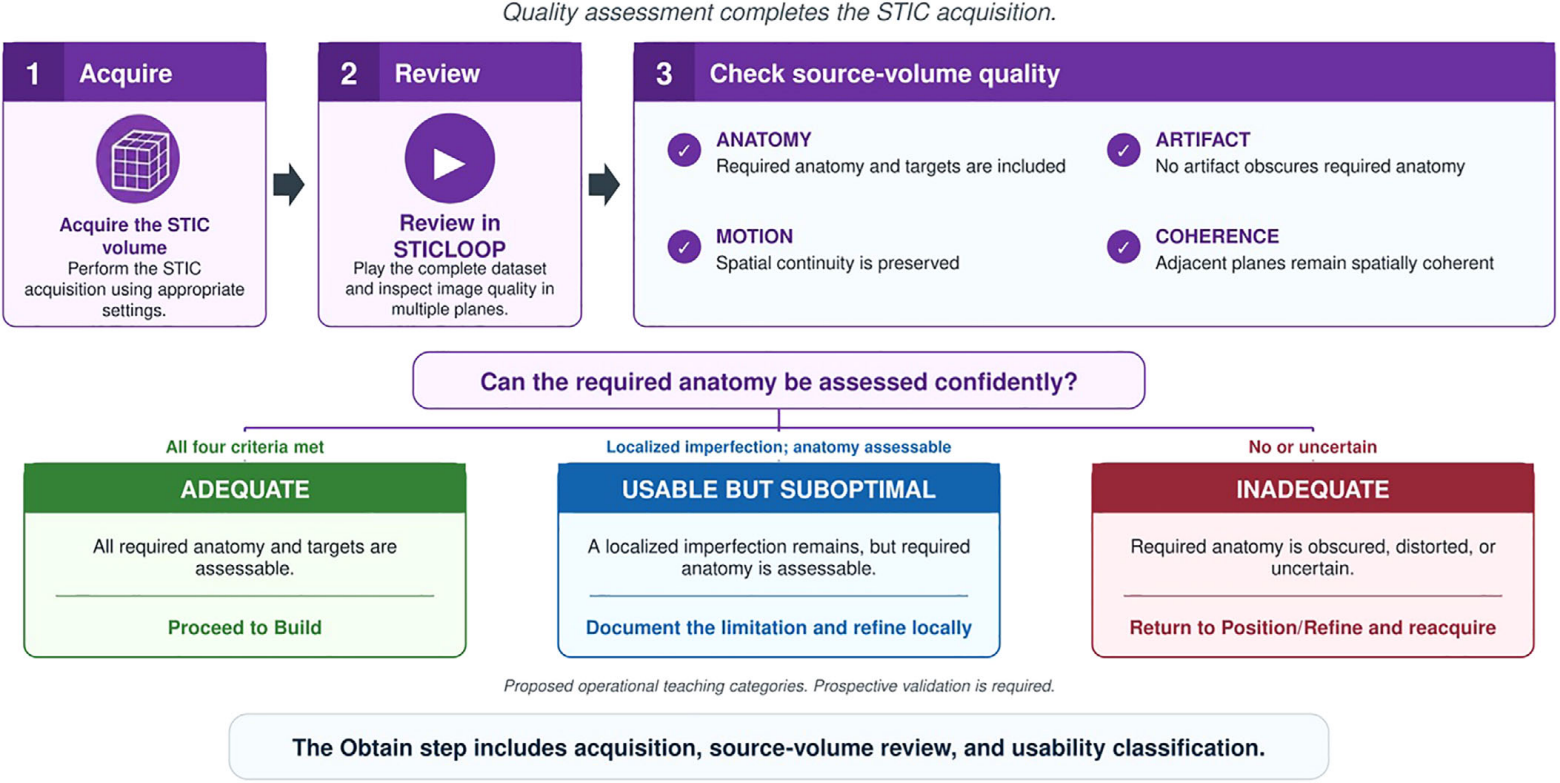
Obtain: STIC volume quality‐control checkpoint. The acquired volume is reviewed in STICLOOP for inclusion of required anatomy and targets, absence of obscuring artifacts, preserved spatial continuity during motion review, and coherence of adjacent planes. The volume is classified as adequate when all criteria are met, usable but suboptimal when a localized imperfection remains but required anatomy is assessable, or inadequate when required anatomy or targets are obscured, distorted or uncertain. Adequate volumes proceed to Build; suboptimal volumes require documentation of the limitation and local refinement; inadequate volumes require reassessment of Position/Refine and reacquisition. These proposed operational teaching categories require prospective validation. Rib‐arc continuity, when used to assess coherence, is a teaching aid and has not been independently validated as an adequacy criterion.

A technically adequate volume includes all required anatomy and targets, without obscuring artifacts or relevant loss of spatial continuity. A volume is considered usable but suboptimal when a localized, recognized imperfection remains but all required anatomy is consistently assessable; the limitation should be documented and local refinement attempted. If required anatomy or targets are obscured, distorted or uncertain, the volume is considered inadequate and should be rejected and reacquired when feasible after reassessment of Position and Refine. These operational teaching categories are proposed for PROBE and require prospective validation (Figure 2; Supplementary Form S1).

*Key message*: A technically adequate STIC volume is the foundation for reliable navigation.

### 2.4 B—Build

Objective: Orient the volume and construct intelligent navigation through anatomically reliable landmark placement.

During Build, the dataset is opened in FINE and its orientation is confirmed before the seven Anatomic Box landmarks are identified: (1) cross‐section of the aorta at the level of the stomach; (2) cross‐section of the aorta at the four‐chamber level; (3) crux cordis; (4) right atrial wall; (5) pulmonary valve; (6) cross‐section of the superior vena cava; and (7) transverse aortic arch [5]. These landmarks are used to generate an internal geometric model and the nine standardized echocardiographic views. Figure 3 illustrates the seven marking planes and the decision cycle applied before each landmark.

**FIGURE 3:**
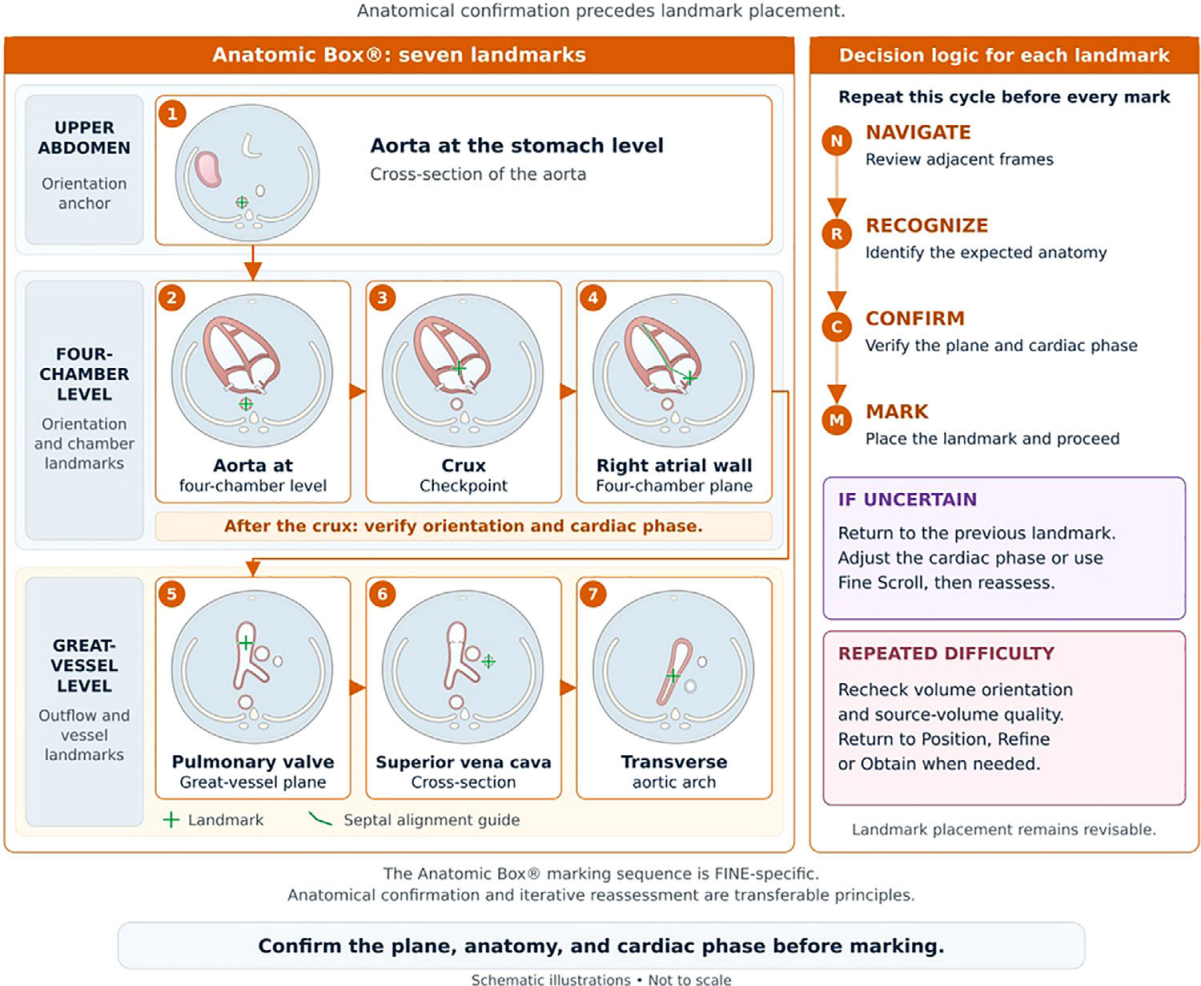
Build: seven‐landmark sequence and decision logic. The anatomical schematics show the Anatomic Box® marking sequence: (1) cross‐section of the aorta at the stomach level; (2) cross‐section of the aorta at the four‐chamber level; (3) crux cordis; (4) right atrial wall; (5) pulmonary valve; (6) cross‐section of the superior vena cava; and (7) transverse aortic arch. Green crosses indicate the marking sites. At the right atrial wall, the green guide indicates alignment with the interventricular septum and ends at the wall marker. For every landmark, the operator navigates, recognizes the anatomy, confirms the plane and cardiac phase, and then marks. Orientation and cardiac phase are reassessed after the crux. Uncertainty prompts local reassessment; repeated difficulty prompts review of source‐volume quality and return to Position, Refine or Obtain when needed. The schematics are not to scale. The marking sequence is specific to FINE; anatomical confirmation and iterative reassessment are transferable teaching principles.

Teaching Build should emphasize anatomical confirmation rather than completion of a predetermined sequence of clicks. Landmark placement is revisable. When the proposed plane, landmark location or cardiac phase is suboptimal, the operator may return to a preceding marking step, navigate through adjacent frames, adjust the cardiac phase when appropriate and reposition the landmark before continuing. Cardiac‐phase recognition is incorporated into FINE to facilitate anatomical marking and improve the success of obtaining cardiac views [6] (Figure 3).

An unexpected discrepancy between the proposed marker position and the expected anatomy should prompt reassessment of volume orientation before the sequence is continued. Reorientation tools may be used when available. Video S3 demonstrates volume reorientation and landmark placement, including confirmation of the crux, right atrial wall, pulmonary valve, superior vena cava and transverse aortic arch. Dynamic playback or fine scrolling can be used when a landmark is not confidently visualized in the initially proposed plane.

The educational emphasis is therefore on a repeated sequence of navigate, recognize, confirm and mark. Difficulty identifying a landmark despite local refinement may reflect a problem originating in Position, Refine or Obtain rather than an isolated marking error.

*Key message*: Accurate landmark placement depends on anatomical confirmation of both plane and cardiac phase.

### 2.5 E—Evaluate

Objective: Critically assess the reconstructed views and refine individual diagnostic planes when additional interrogation is required.

Automatic generation of the nine diagnostic planes does not end the FINE workflow. Each reconstructed plane remains available as a navigable volume and should be reviewed for anatomical consistency, technical quality and relevant acquisition or reconstruction artifacts [5, 6].

Apparent anatomical discontinuities or altered spatial relationships may reflect true pathology or acquisition/reconstruction artifacts. Motion during STIC acquisition, spatial misregistration or an imperfect reconstructed plane may produce artifactual appearances. Navigation through the dataset can help assess whether a questionable appearance is related to acquisition or reconstruction and whether the original volume remains technically adequate for further analysis (Figure 4). Any suspicious finding that persists after plane interrogation, or cannot be confidently attributed to a recognized artifact, requires verification with real‐time two‐dimensional ultrasound and color Doppler, with referral for fetal echocardiography when indicated [1]. If verification cannot be completed, the finding remains unresolved and must not be accepted as normal.

**FIGURE 4:**
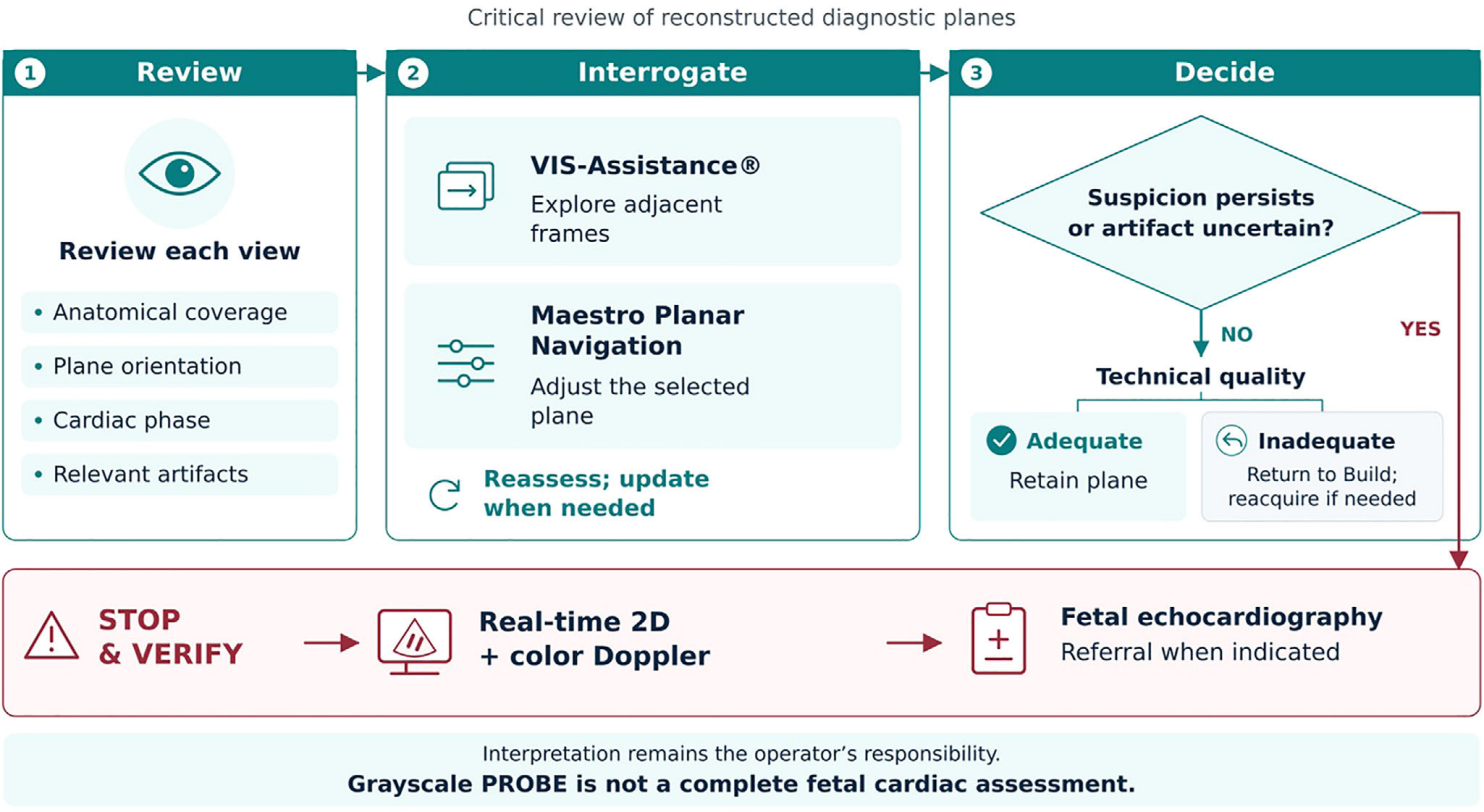
Evaluate: critical review of reconstructed diagnostic planes. Each view is reviewed for anatomical coverage, plane orientation, cardiac phase and relevant artifacts. Navigation with VIS‐Assistance or Maestro Planar Navigation is used, as appropriate, to interrogate the plane and surrounding anatomy. Following adjustment, the plane is reassessed and updated when appropriate. A technically inadequate plane prompts review of landmark placement and source‐volume quality, with reacquisition when needed. Any suspicious finding that persists after plane interrogation, or cannot be confidently attributed to a recognized artifact, requires verification with real‐time 2D ultrasound and color Doppler, with referral for fetal echocardiography when indicated. The grayscale PROBE workflow does not constitute a complete fetal cardiac assessment; interpretation remains the operator's responsibility.

### 2.6 VIS‐Assistance

VIS‐Assistance provides automated sonographic navigation around a selected diagnostic plane [5, 6]. The operator can explore adjacent frames with the image frozen or during dynamic playback, compare candidate planes, bookmark selected frames and, when appropriate, update the diagnostic plane with the preferred view. Video S4 demonstrates this workflow using the left ventricular outflow tract as an example.

### 2.7 Maestro Planar Navigation

For advanced local refinement, Maestro Planar Navigation permits independent fine‐tuning of a single FINE‐generated diagnostic plane without altering the other eight planes [6]. A parallel shift (Move View on the system shown) can be attempted first, followed, when necessary, by small rotational corrections around the X, Y and Z axes. Yeo and Romero recommend attempting parallel‐shift movements before rotational movements because a small change in plane depth may be sufficient to improve visualization [6].

Video S4 also demonstrates review of the bicaval plane in a technically usable but suboptimal volume containing localized motion‐related distortion. Manual navigation allows the superior and inferior vena caval connections with the right atrium to be assessed while the acquisition artifact remains explicitly recognized. This example illustrates why Evaluate should distinguish between a local artifact, a plane that requires refinement and a dataset that is truly inadequate.

VIS‐Assistance and Maestro Planar Navigation are complementary: the former provides automated dynamic exploration around a diagnostic plane, whereas the latter permits targeted operator‐controlled translational and rotational refinement of an individual reconstructed plane.

*Key message*: Automation generates the diagnostic planes; critical review and appropriate refinement remain operator responsibilities.

## 3 Probe as An Iterative Workflow

Although the acronym is presented linearly as P → R → O → B → E, its application is not rigidly unidirectional.

Quality assessment at each stage functions as a decision point. Not every imperfection detected during Evaluate requires complete reconstruction or reacquisition: some planes can be corrected locally using VIS‐Assistance or Maestro Planar Navigation.

When local refinement is insufficient, the operator should trace the problem backward through the workflow. A problem detected during Evaluate may require returning to Build; an unreliable landmark during Build may reveal an inadequate dataset and require returning to Obtain; and repeatedly unsatisfactory acquisition may require a new Position or Refine. Landmark placement itself can contain a short iterative loop in which the operator returns to a previous landmark, optimizes the plane or cardiac phase and repeats the marking before completing the Anatomic Box. Video S5 demonstrates this return process.

This return logic differentiates PROBE from a purely operational checklist. When a technically usable volume is suboptimal, the operator should identify where quality was lost, refine what is correctable and use that information to inform subsequent acquisition and navigation. The aim is structured learning from real‐world datasets, not the assumption that every clinical STIC volume will be perfect.

*Key message*: If quality is insufficient, return to the step where the problem originated.

## 4 Discussion

PROBE organizes established imaging principles into explicit teaching decisions, including when to return to an earlier step. Table 2 distinguishes this educational organization from DAZE, the 5‐4‐3‐2‐1 method and FINE volume‐adequacy criteria [5, 7, 12, 13]. The uppercase mnemonic denotes the workflow; “transducer” denotes the ultrasound device. FINE studies address volume adequacy, view generation and abnormal anatomy [7, 8, 9, 14, 15]; their results do not establish PROBE's educational effectiveness.

**TABLE 2:** Scope and reported evidence of approaches relevant to PROBE.

Approach	Target in the imaging process	Modality	Relationship to PROBE	Evidence / validation in cited report(s)
**DAZE** [12]	Image optimization during scanning: depth, insonation/sector angle, zoom and system settings.	Real‐time 2D; color Doppler.	Overlaps Position and Refine.	Technical teaching article; no dataset generated or analyzed. The report does not establish educational effectiveness.
**5‐4‐3‐2‐1** [13]	Structured anatomical screening: overview, four‐chamber, great‐vessel and outflow checks, followed by a Doppler check.	Real‐time 2D; color Doppler.	Organizes screening checks across views; PROBE organizes technical use of STIC/FINE.	Method description without study data; no prospective validation reported in that article.
**FINE volume adequacy criteria** [5, 7]	STIC acquisition and selection: coverage, spine orientation, shadowing, motion and spatial consistency.	Volumetric STIC reviewed before FINE processing.	Provides established adequacy principles underlying Obtain.	Applied in FINE development/testing and a prospective evaluation. These studies assess FINE view generation, not an independent educational validation of the adequacy criteria.
**PROBE (present article)**	Links acquisition preparation, volume review, landmark confirmation and review of reconstructed planes.	Grayscale 2D preparation and STIC/FINE.	Integrates established principles into five teaching steps, return decisions and Supplementary Form S1.	Proposed educational framework; no formal teaching cohort or prospective validation. Form S1 is also unvalidated.

Comparison is limited to the cited publications; it is not a systematic review. PROBE organizes established principles for teaching, without a claim of technical novelty or superior performance.

Abbreviations: 2D, two‐dimensional; DAZE, Depth, Angle, Zoom, Enhancement; FINE, Fetal Intelligent Navigation Echocardiography; PROBE, Position, Refine, Obtain, Build, Evaluate; STIC, spatio‐temporal image correlation.

On STIC systems with suitable datasets and manual multiplanar navigation, including compatible Samsung systems without FINE/5D Heart, Position, Refine and Obtain retain preparation, optimization and volume review. Build may involve orientation and manual reconstruction of individual views, while Evaluate retains anatomical review and refinement [4, 9]. Anatomic Box, VIS‐Assistance and Maestro Planar Navigation are FINE‐specific [5, 6]. This proposed educational transfer remains untested; Form S1 addresses FINE.

PROBE derives from the first author's longstanding informal teaching of physicians during clinical applications. It has not been delivered as a formal program under the PROBE name, and no learner cohort, numbers by experience level or educational outcomes are reported. PROBE, Form S1 and practical cues such as rib‐arc continuity and landmark position remain unvalidated.

Supplementary Form S1 anchors assessment of all five steps and distinguishes learner performance from source‐volume quality. Ratings are Independent, Prompted, Unmet and Not observed. The form is proposed for formative assessment, not certification; neither its criteria nor its non‐compensatory completion rule has been validated.

For prospective validation, the proposed primary outcome is the proportion of initiated standardized post‐training assessments completed independently: all five steps rated Independent and the safety decision demonstrated without coaching. One assessment per learner would combine observed acquisition and prespecified volume‐review tasks; a suitable dataset could support Build and Evaluate after rejection of an inadequate volume. Safety may be assessed through a standard scenario. Incomplete assessments count as non‐completions, with their number and reasons reported separately. Future studies should assess rater calibration, interobserver agreement and multicenter reproducibility; secondary outcomes may include volume adequacy, reacquisition, completion time and retention.

This grayscale workflow supports technical training and does not constitute a complete cardiac assessment. Color and power Doppler acquisition are outside its scope [16]. Suspicious or indeterminate findings require the real‐time 2D and color Doppler verification and referral pathway described in Evaluate [1]. Clinical interpretation remains the responsibility of appropriately qualified clinicians.

## 5 Conclusion

The PROBE Educational Workflow organizes the teaching of FINE into five sequential and interdependent steps: Position, Refine, Obtain, Build and Evaluate. Its purpose is to shift the focus from simple software operation to understanding the entire technical process that supports volumetric navigation, from image preparation to quality control of reconstructed views. PROBE does not modify the FINE algorithm, does not constitute a diagnostic protocol and does not replace formal training in fetal echocardiography. It offers a simple educational framework to guide acquisition, navigation and error recognition. Prospective validation should determine its impact on learning, reproducibility and training efficiency.

## Funding

The authors have nothing to report.

## Ethics Approval

Our investigations were carried out following the rules of the Declaration of Helsinki of 1975, revised in 2013. As this article is a practice one, it was not necessary ethics committee approval.

## Consent

Patients gave their consent for the figure's publication.

## Conflicts of Interest

The authors declare no conflict of interest.

## Supporting information




**Supporting Information: Video S1**. Position and Refine. Demonstration of the preparation of the two‐dimensional image before STIC acquisition. The operator identifies fetal position and the acoustic window, selects the fetal echocardiography preset, centers the heart, performs a brief cranial sweep from the four‐chamber view toward the outflow tracts to check for rib shadowing over the great vessels, optimizes depth, gain and frequency when necessary, activates 5D Heart, adjusts the volume box and initiates STIC acquisition (53.68 s; 15.65 MB).

**Supporting Information: Video S2**. Obtain: STIC acquisition and STICLOOP quality review. The STIC acquisition is completed and the dataset enters STICLOOP for quality assessment. Automatic playback is paused and the acquisition planes are reviewed manually to assess spatial coherence. Rib‐arc continuity and alignment are used as practical visual cues to prompt closer review for obliquity, misalignment or motion‐related distortion before the volume is accepted for FINE processing. These visual cues are teaching aids and have not been independently validated as adequacy criteria (57.76 s; 7.80 MB).

**Supporting Information: Video S3**. Build: volume orientation and Anatomic Box landmark placement. Demonstration of volume orientation and the seven Anatomic Box landmarks. An unexpected relationship between the proposed marker and the expected crux prompts reassessment and volume reorientation. Landmark placement is then continued with anatomical and cardiac‐phase confirmation; dynamic playback or fine scrolling is used when needed before marking the pulmonary valve, superior vena cava and final transverse aortic arch landmark. Completion of the sequence generates the nine diagnostic planes (105.04 s; 11.59 MB).

**Supporting Information: Video S4**. Evaluate: VIS‐Assistance and Maestro Planar Navigation. The nine FINE‐generated diagnostic planes are demonstrated as navigable volumes. A left ventricular outflow tract plane is selected for VIS‐Assistance, with frozen and dynamic navigation, bookmarking, comparison of candidate frames and update of the diagnostic plane. Maestro Planar Navigation is then demonstrated using Move View/parallel shift and X‐, Y‐ or Z‐axis rotation. A bicaval‐plane example shows localized motion‐related distortion in a technically usable but suboptimal volume while the superior and inferior vena caval connections to the right atrium remain assessable. Any suspicious finding that persists after plane interrogation, or cannot be confidently attributed to a recognized artifact, requires verification with real‐time two‐dimensional ultrasound and color Doppler, with referral for fetal echocardiography when indicated. The grayscale PROBE workflow does not constitute a complete fetal cardiac assessment (126.16 s; 33.85 MB).

**Supporting Information: Video S5**. Iterative workflow: return and landmark revision. Demonstration of the iterative nature of PROBE. During Anatomic Box landmark placement, the operator returns to a previous step when refinement is needed, reassesses the plane and landmark, and proceeds only after the marking has been improved. The example illustrates the core PROBE principle: when quality is insufficient, return to the step where the problem originated (27.88 s; 1.54 MB).

**Supporting Information: Supplementary Form S1**. PROBE educational assessment. One‐page observer‐rated form with performance anchors for Position, Refine, Obtain, Build and Evaluate; separate source‐volume classification; a safety decision; and an explicit rule for independent completion. The form is proposed for supervised teaching and prospective evaluation and has not been validated.

## Data Availability

Data sharing is not applicable to this descriptive educational article because no research dataset was generated or analyzed.
