# Supplementary material for: A Structured Approach to Teaching Volumetric Fetal Echocardiography Using Fetal Intelligent Navigation Echocardiography (FINE)
Source: Echocardiography. 2026 Oct 31;43(11):e70659. doi: 10.1111/echo.70659 (PMC13723304; doi:10.1111/echo.70659)
Supplement: Supplementary file 1 [file ECHO-43-e70659-s006.docx]

**Supplementary Form S1**

**PROBE educational assessment**

Observer-rated teaching form for grayscale FINE. Proposed criteria require prospective validation.

Learner ID __________ FINE experience (years) _____ Assessor ____________ Date __________

Case / volume ID(s) ______________ Gestation (weeks + days) ______ Platform ______________

**Session** ☐ Baseline	☐ After training	☐ Follow-up	Acquisition attempts _____

Use anonymized identifiers. Observe P–E; if a volume is rejected, use a suitable dataset to assess Build and Evaluate. Rate the decisions, not image perfection. Select one rating per step.

I Independent — all listed criteria met without cues. P Prompted — met after verbal coaching without takeover.
U Unmet — criteria unmet, assessor takeover or unsafe progression. NO Not observed — record the reason.

| **Step** | **Observable performance criteria** | **Rating** |
| --- | --- | --- |
| **P Position** | Identifies fetal position, an axial four-chamber plane and an acoustic window; recognizes shadowing and adjusts the approach or defers acquisition when needed. | ☐ I ☐ P ☐ U ☐ NO |
| **R Refine** | Selects a cardiac preset; optimizes depth, magnification, focus, gain and frequency while preserving interfaces and temporal resolution; checks the region of interest before acquisition. | ☐ I ☐ P ☐ U ☐ NO |
| **O Obtain** | Adapts sweep angle/time, holds the transducer steady and reviews the entire volume for coverage from stomach to upper mediastinum, shadowing, motion and spatial coherence. Classifies the volume below and selects the next action. | ☐ I ☐ P ☐ U ☐ NO |
| **B Build** | Confirms orientation and phase, rechecks after crux and marks seven targets: aorta at stomach; aorta at four-chamber level; crux; right atrial wall with septal alignment; pulmonary valve; superior vena cava; transverse aortic arch. Revises uncertain marks or returns to Obtain. | ☐ I ☐ P ☐ U ☐ NO |
| **E Evaluate** | Reviews all nine reconstructed views for coverage, orientation, phase and artifacts; selects local refinement or return to the source step and applies the safety rule below. | ☐ I ☐ P ☐ U ☐ NO |

**Source volume classification and next action**

☐ **Adequate —** Required anatomy/targets assessable; no obscuring artifact or relevant spatial discontinuity. Proceed to Build.

☐ **Usable but suboptimal —** Localized, recognized imperfection; all required anatomy remains assessable. Document and refine locally.

☐ **Inadequate —** Required anatomy/targets missing, obscured, distorted or uncertain. Reject; reassess Position/Refine and reacquire when feasible.

Volume ID for this classification __________ Limitation / action ___________________________________

**Safety decision**

Persistent suspicion after plane interrogation, or uncertainty about artifact, requires real-time 2D and color Doppler verification and referral for fetal echocardiography when indicated. Unverified findings remain unresolved and must not be accepted as normal.

**Independently demonstrated** ☐ Yes	☐ No	☐ Not observed (Use a standard scenario if needed.)

**Assessment outcome and feedback**

**Independent completion demonstrated** ☐ Yes	☐ No

Yes requires I in all five steps and an independently demonstrated safety decision. Any P, U or NO means No. Do not sum ratings; record incomplete observations and their reasons.

Feedback / reason for incomplete observation __________________________________________________
